# The influence of non-DNA-targeted effects on carbon ion–induced low-dose hyper-radiosensitivity in MRC-5 cells

**DOI:** 10.1093/jrr/rrv072

**Published:** 2015-11-10

**Authors:** Fei Ye, Jing Ning, Xinguo Liu, Xiaodong Jin, Tieshan Wang, Qiang Li

**Affiliations:** 1 Medical Physics Division, Institute of Modern Physics, Chinese Academy of Sciences, Lanzhou 730000, China; 2 Department of Modern Physics, Lanzhou University, Lanzhou 730000, China; 3 University of Chinese Academy of Sciences, Beijing 100049, China; 4 Key Laboratory of Heavy Ion Radiation Biology and Medicine of Chinese Academy of Sciences, Lanzhou 730000, China; 5 Gansu Provincial People's Hospital, Lanzhou 730000, China

**Keywords:** low-dose hyper-radiosensitivity, non-DNA-targeted effect, gap junctional intercellular communication, carbon-ion radiation

## Abstract

Low-dose hyper-radiosensitivity (LDHRS) is a hot topic in normal tissue radiation protection. However, the primary causes for LDHRS still remain unclear. In this study, the impact of non-DNA-targeted effects (NTEs) on high-LET radiation–induced LDHRS was investigated. Human normal lung fibroblast MRC-5 cells were irradiated with high-LET carbon ions, and low-dose biological effects (in terms of various bio-endpoints, including colony formation, DNA damage and micronuclei formation) were detected under conditions with and without gap junctional intercellular communication (GJIC) inhibition. LDHRS was observed when the radiation dose was <0.2 Gy for all bio-endpoints under investigation, but vanished when the GJIC was suppressed. Based on the probability of cells being hit and micro-dose per cell calculation, we deduced that the LDHRS phenomenon came from the combined action of direct hits and NTEs. We concluded that GJIC definitely plays an important role in cytotoxic substance spreading in high-LET carbon ion–induced LDHRS.

## INTRODUCTION

Low-dose hyper-radiosensitivity (LDHRS) is a phenomenon in which irradiated cells exhibit higher radiosensitivity than that predicted by the extrapolation from high-dose responses, and it has been widely reported for low and high linear-energy-transfer (LET) radiations [1–3]. Due to technical advances in radiotherapy, especially the application of intensity-modulated radiation therapy (IMRT), a greater volume of normal tissues surrounding target volumes are irradiated at low doses per fraction. Concerns about normal tissue damage due to low-dose irradiation have been raised [4–6]. Therefore, LDHRS, as an important issue in the radiation protection of normal tissues, needs to be thoroughly investigated.

For low-LET radiations, LDHRS is followed by increased radioresistance (IRR). DNA repair and cell cycle checkpoints are two of the main research areas in this field. Many experiments have suggested that the activation of DNA repair processes is important for overcoming LDHRS and for the development of IRR. The roles of non-homologous end joining [7], DNA-dependent protein kinase activity [8], homologous recombination [9] and mismatch repair [10] in LDHRS and IRR have been identified in last two decades. Another popular hypothesis is that LDHRS is mainly observed in cells in the G_2_ phase of the cell cycle, and a novel early G_2_/M checkpoint activated in the dose range of 0–40 cGy contributes to LDHRS significantly. These experimental data were reviewed by Marples [11]. Apart from the damage caused by radiation directly, radiation-induced bystander effects are believed to be another possible way to induce LDHRS. Using the medium transfer method and γ-rays, Nuta *et al.* [12] observed the phenomenon of LDHRS in the bystander cells of BJ human foreskin fibroblasts.

Although progress in understanding low-dose high-LET-radiation–induced LDHRS has been made in recent years, the difference between LDHRS induced by low- and high-LET radiations is still largely unknown. The similarity of the mechanisms by which LDHRS is induced by low- and high-LET radiations was reviewed by Heuskin *et al.* [13]. Compared with their responses to conventional low-LET radiations, cells have unique responses to high-LET radiations, such as less cell-cycle dependence [14]. Moreover, the probability of cells not being hit by particles can be calculated for any doses. The non-DNA-targeted effects (NTEs) of ionizing radiations are defined as the responses triggered by radiation energy deposition in cellular targets other than nuclear DNA, including genomic instability, adaptive responses and bystander effects, and they are currently considered to be a candidate mechanism for radiation risk at low doses [15].

This study aimed to demonstrate the influence of high-LET carbon-ion radiation–induced NTEs on LDHRS using a normal human lung fibroblast cell line. Cell hit probability was calculated in order to determine the proportion of nuclei unhit cells. To reveal the role of gap junctional intercellular communication (GJIC) in high-LET radiation-elicited LDHRS, GJIC was suppressed, using its specific inhibitor. Our hope was, through this study, to provide clues for understanding the mechanisms for high-LET heavy-ion radiation–induced LDHRS.

## MATERIALS AND METHODS

### Cell culture and cell area determination

Normal human lung fibroblast MRC-5 cells were cultured in DMEM medium (Hyclone, USA) supplemented with 10% fetal bovine serum (Hyclone, USA), 100 µg/ml streptomycin and 100 U/ml penicillin (Hyclone, USA) and were incubated at 37°C in a humidified atmosphere of 5% CO_2_. Confluent cells were stained with Hoechst 33342 and photographed with a fluorescence microscope (Olympus DP72, Japan). Outlines of the cells and their nuclei were drawn, and then cell and nucleus areas were measured using ImageJ 1.48 software. All data were represented as mean ± standard deviation (SD).

### Hit probability calculation, microscopic dose estimate and irradiation

Due to the random nature of radiation action on cells and the uniform dose distribution in a radiation field, the hit number of a cell by particle radiation follows a Poisson distribution [16].The microscopic dose (*d*) per traversal to a thin disk-shaped MRC-5 cell can be calculated according to *d* = 0.16 × *LET*/*A* [17], where *A* is the area of the cell, and *LET* is the LET value of the particle. The units for *d*, *LET* and *A* are Gy, keV/μm and μm^2^, respectively.

Cells were exposed to a carbon-ion beam (165 MeV/u), generated by the Heavy Ion Research Facility in Lanzhou (HIRFL) at the Institute of Modern Physics (IMP), Chinese Academy of Sciences, China. During exposure, the dose rate and LET value of the carbon-ion beam were adjusted to be 5 cGy/min and 70 keV/μm, respectively, and the thickness of the energy degrader was set to be 51.1 mm (water-equivalent path length). Dosimetry was carried out using a dosimeter. Cells were cultured in four adjacent wells of 24-well plates and placed at the center of the carbon-ion irradiation field (5 cm × 5 cm), perpendicularly to the beam incident direction. The diameter of each well was 2 cm, and the thickness of the well bottom was 1.3 mm. Under these conditions, the uniformity of the irradiation field was close to 100%. The cell samples were divided into two groups: the radiation-only group (R) and the group receiving co-treatment with radiation and 18-α-glycyrrhetinic acid (R + AGA).

### Inhibition of Gap Junction Communication

AGA (Sigma-Aldrich), a reversible inhibitor of GJIC, was dissolved in dimethyl sulfoxide and added to cell cultures at a concentration of 50 μM at 30 min prior to irradiation. Then, the cells were incubated in the presence of the inhibitor until they were harvested. Using this protocol, AGA did not alter the plating efficiency of unirradiated cells but did inhibit cell coupling. Control cell cultures were incubated with just the dissolving vehicle.

### Functional analysis of GJIC using a scrape-and-scratch method

A scrape-and-scratch method was used to test the inhibition level of GJIC by AGA [18, 19]. Briefly, Lucifer Yellow does not diffuse through membranes, but its low molecular weight permits its transmission from one cell to another, presumably across patent gap junctions. After co-culturing with AGA (50 μM) for 30 min, confluent cells cultured on coverslips were rinsed with phosphate-buffered saline (PBS); next, two longitudinal scratches were created by pipette tips through the cell monolayer. The coverslip was immersed in 0.5% Lucifer Yellow (sigma) for exactly 1 min and then rinsed three times with PBS. Finally, the cells were examined by means of fluorescence microscopy. The area of the dye transferred from the scratched margin of the AGA-treated cells was measured using ImageJ 1.48 software and compared with that of cells without AGA treatment.

### Phospho-histone γH2AX foci formation

Autsavapromporn *et al.* [20] reported that co-culture time after irradiation is essential in order to observe LDHRS. Therefore, despite the maximum number of γH2AX foci being observed at 30 min after irradiation, the cell harvest time in this study was determined to be 4 h after irradiation. In addition, because only 2D images could be acquired, the method of using fluorescence microscopy to count the number of γH2AX foci (distributed in 3D space) in cells would introduce experimental error. For better describing the LDHRS phenomenon, an improved calculation method was applied in this study. Instead of counting the mean γH2AX foci number per cell, the ratio of cells with γH2AX foci to all counted cells was used.

Four hours after irradiation, cells were washed in cold PBS and fixed for 15 min in 4% w/v paraformaldehyde. The cells were then permeabilized for 10 min in 0.3% v/v Triton X-100 (Sigma-Aldrich) in PBS, and washed twice with PBS. Antibodies were diluted with 1% w/v bovine serum albumin in PBS. Next the cells were incubated with rabbit anti-γH2AX antibody (CST) for 2 h at 37°C, washed three times with PBS and incubated with fluorescein isothiocyanate-conjugated anti-rabbit IgG antibody (Santa Cruz Biotechnology) for 1.5 h at room temperature. The slides were incubated in PBS containing DAPI (4′,6-diamidino-2-phenylindole, 0.5μg/ml) for 10 min so as to stain the DNA. Then the samples were photographed with a fluorescence microscope (Olympus, DP72, Japan). After more than 10 000 cells were counted, the ratio of cells with γH2AX foci to all counted cells was calculated.

### Micronuclei formation

The frequency of micronuclei formation was measured with the cytokinesis block technique. Four hours after irradiation, confluent cells were subcultured and ∼3 × 10^4^ cells were seeded in a Petri dish in the presence of 2 μg/ml cytochalasin B (Sigma-Aldrich) and then incubated at 37°C in an incubator. After 72 h, the cells were rinsed with PBS, fixed in ethanol, stained with Hoechst 33342 solution (1μg/ml PBS), and then viewed under a fluorescence microscope (Olympus, DP72, Japan). At least 1000 binucleated cells were examined, and only micronuclei in binucleated cells were considered for analysis. Cytochalasin B at the concentration mentioned above was not toxic to MRC-5 cells.

### Colony-formation assay

A colony-formation assay was performed at 4 h post-irradiation. Briefly, after trypsinization and replating, cells were incubated for 2 weeks. Then the cell colonies were fixed with ethanol, stained with Coomassie brilliant blue and then counted using a stereomicroscope. A colony with >50 cells was scored as a ‘survivor’. The cell survival fraction (SF) was normalized to the sham-irradiated control.

### Statistical analysis

Two independent experiments were performed in triplicate. The student's *t*-test of independent samples was conducted using SPSS software (version 22) at the same dose. Differences were considered significant when *P* < 0.05.

## RESULTS

**Fig. 1.**
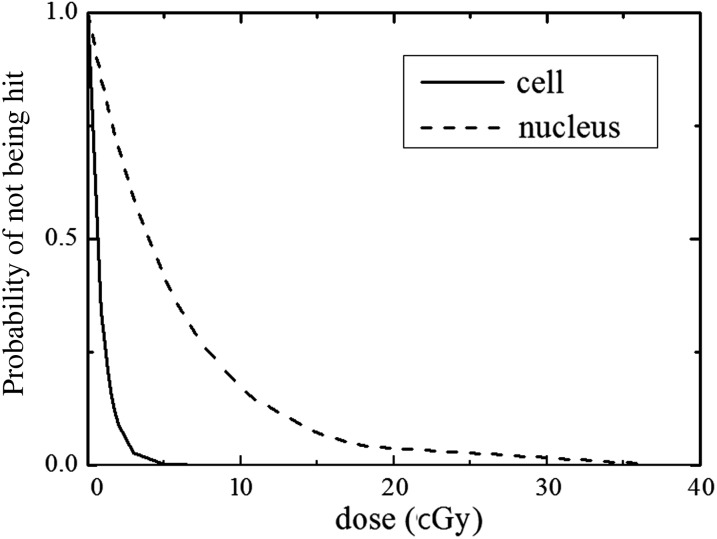
The probability of MRC-5 cells not being hit by carbon ions was calculated according to the hit number in a cell by particle radiation, which follows a Poisson distribution.

The mean areas of MRC-5 cells and nuclei were determined to be (1.30 ± 0.51) × 10^-5^ cm^2^ (*n* = 50) and (1.96 ± 0.53) × 10^-6^ cm^2^ (*n* = 50), respectively. Therefore, the probabilities for cells and their nuclei not being hit by carbon ions were calculated, and the results are shown in Fig. 1 and Table 1.

**Table 1.** The probabilities of cells and nuclei not being hit at different macroscopic absorbed doses

Dose (cGy)	Probability of not being hit	
	Cell	nucleus
0.8	0.381	0.868
1.5	0.163	0.768
7.5	0.001	0.266
18	2.43 × 10^-10^	0.042
37	4.95 × 10^-20^	1.46 × 10^-3^
100	1.06 × 10^-52^	2.19 × 10^-8^
200	4.44 × 10^-105^	4.78 × 10^-16^

**Fig. 2.**
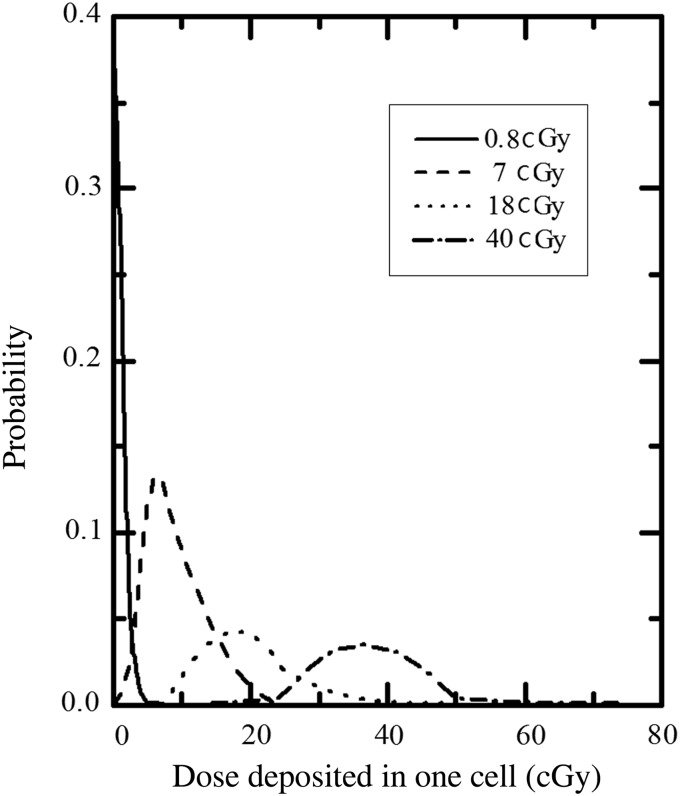
The microscopic distribution of doses deposited in one MRC-5 cell by carbon ion traversals at a certain macroscopic absorbed dose.

The microscopic dose in a cell per traversal was 0.83 cGy. For a certain macroscopic dose, the microscopic dose in a single cell varied over a relatively large range. For example, the microscopic dose distribution was from 10 to 40 cGy when the macroscopic dose was 18 cGy (Fig. 2).

**Fig. 3.**
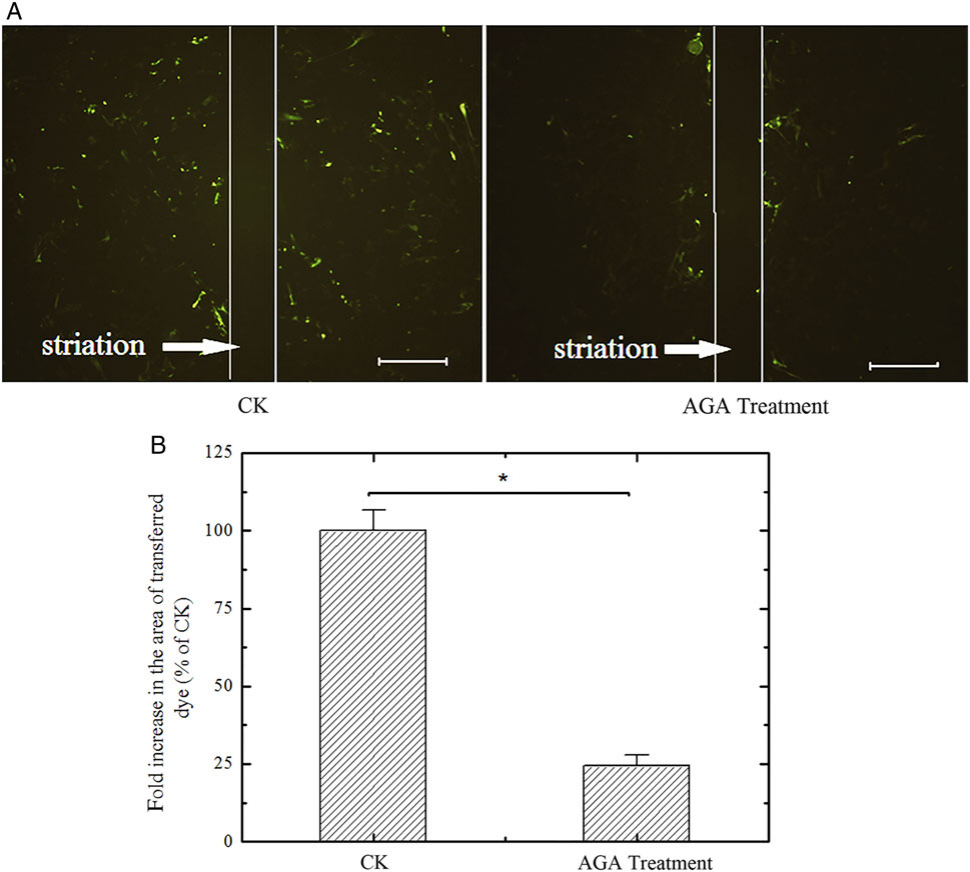
(**A**) Fluorescence photomicrographs of scrape/scratch experiments using Lucifer Yellow. Gap junction intercellular communication (GJIC) was blocked in MRC-5 cells treated with 30 min of 18α-glycyrrhetinic acid (50 μM). Bar: 500 μm. (**B**) The percentage of Lucifer Yellow transferred area of 18α-glycyrrhetinic acid–treated MRC-5 cells was 24.40 ± 3.43%. The GJIC of confluent cultured MRC-5 cells was significantly blocked by 18α-glycyrrhetinic acid (*P* < 0.05, *n* = 10).

AGA was used to inhibit GJIC in this study, and the inhibition level was measured with the scrape-and-scratch method. In the control experiment, scrape-loaded cells in the presence of Lucifer Yellow showed positive transfer of Lucifer Yellow between cells. In contrast, cells treated with AGA appeared to lose the ability to communicate with each other (Fig. 3A), and the dye transfer was blocked to 24.40 ± 3.43% of the intensity compared with the control group (*P* < 0.05, *n* = 10) (Fig. 3B).

**Fig. 4.**
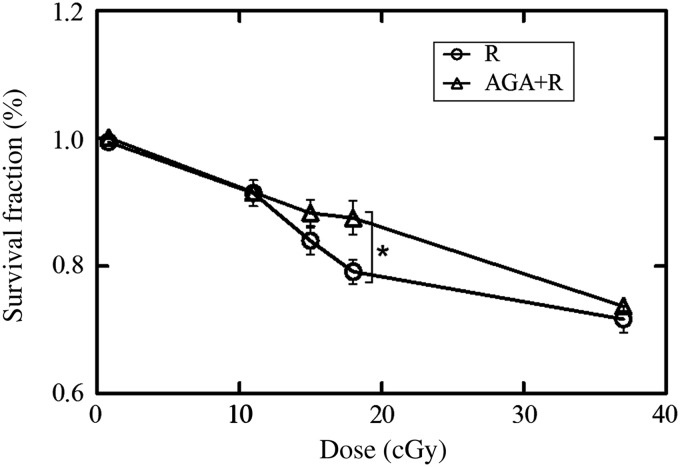
Cell survival curves derived from the colony-formation assay. The survival fractions were statistically different at 18 cGy between the R and R + AGA groups (*P* = 0.016).

In Fig. 4 the survival fractions of MRC-5 cells under the various conditions are shown. In the R group, the LDHRS phenomenon was observed in the low-dose region (Fig. 4). Carbon ion radiation of 18 cGy led to a significantly lower survival fraction than in the R + AGA group (*n* = 6, *P* = 0.016). At the dose of 18 cGy, the probabilities of cells and nuclei not being hit by carbon ions were calculated to be 2.40 × 10^-10^ and 4.18 × 10^-2^, respectively, as shown in Table 1.

**Fig. 5.**
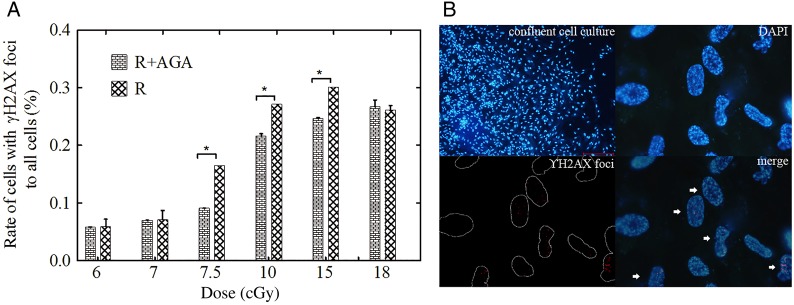
(**A**) The ratio of cells with phospho-histone γH2AX foci to all cells was measured at 4 h after irradiation. In the dose range of 7.5–15 cGy, the rate of cells with γH2AX foci was significantly higher in the R group than in the R + AGA group (*n* = 6, *P* < 0.05 at 7.5 cGy; *n* = 6, *P* < 0.05 at 9.5 cGy; *n* = 6, *P* < 0.05 at 15 cGy). (**B**) The immunofluorescence photograph of confluent cultured MRC-5 cells, DAPI staining of MRC-5 cells and γH2AX foci formation in irradiated MRC-5 cells.

**Fig. 6.**
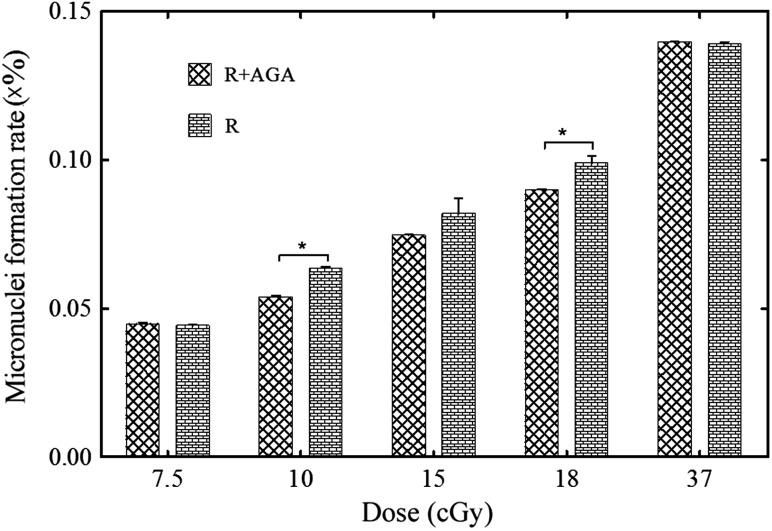
Micronuclei formation rates for the various treatments (R, R + AGA) at various doses. In the dose range of 10–18 cGy, the micronuclei rate in binucleated cells was higher in the R group than in the R + AGA group (*n* = 6, *P* < 0.05 at 10 cGy; *n* = 6, *P* = 0.065 at 15 cGy; *n* = 6, *P* < 0.05 at 18 cGy).

The number of cells with persistent γH2AX foci was measured by counting the cells with γH2AX foci in the nuclei (Fig. 5). In the dose range of 7.5–15 cGy (Fig. 5A), the ratio of cells with γH2AX foci to all counted cells was obviously higher in the R group than in the R + AGA group (*n* = 6, *P* < 0.05 at 7.5 cGy; *n* = 6, *P* < 0.05 at 9.5 cGy; *n* = 6, *P* < 0.05 at 15 cGy). The probability of cells not being hit by the carbon ions was calculated to be 1.21 × 10^-4^ to 1.45 × 10^-8^ in the dose range of 7.5–15 cGy (Table 1). A similar phenomenon was observed using micronuclei formation as the bio-endpoint (Fig. 6). In the dose range of 10–18 cGy, the micronuclei formation rate was significantly higher in the R group than in the R + AGA group (*n* = 6, *P* < 0.05 at 10 cGy; *n* = 6, *P* = 0.065 at 15 cGy; *n* = 6, *P* < 0.05 at 18 cGy).

## DISCUSSION

According to the probability of not being hit calculation, it could be clearly seen that LDHRS was observed in the dose range when nearly all cells were hit by carbon ions. Hence, there was almost no bystander effect (radiation-induced cytotoxicity factor spreading from hit cells to cells that haven't been hit). However, a number of nuclei that had not been hit still existed in this dose range, and the LDHRS was eliminated by inhibition of GJIC. These results suggest that there was a synergistic action between DNA-targeted effects and NTEs on cell inactivation, and GJIC was the major method of mediating the LDHRS. In addition, according to the study by Fournier *et al.* using microbeams [21], the probability of a cell surviving the nucleus traversal by exactly one carbon ion was 25%, significantly lower than that in the case of broad beam, in which the mean number of particle traversals per nucleus was computed to be 1. Collectively, the lethal effect induced by the direct hit of nuclei by carbon ions predominated when the dose was high; however, the GJIC-mediated NTEs contributed a lot to the overall response in the low-dose region. Notice that both the experimental measurements and the theoretical calculations have been discussed in this study. Although the uniformity of the irradiation field of the broad beam was close to 100%, the theoretical calculation of the probability of the nucleus not being hit (according to the nature of particle radiation action) could not describe the actual situation accurately. This might be a limitation of our present study. For further elaborate studies, heavy-ion microbeams should be used.

In principal, there are two directions in which toxic substances from the NTEs can spread between cells. One is from the irradiated to the unirradiated cells; another is from the unirradiated to the irradiated cells. Which way would have the more lethal effect is still unknown. However, an increase in dose results in a decrease in the number of cells that provide or receive lethal cytotoxic substances, thereby reducing NTE-induced cell death, ultimately causing the various resultant bio-endpoints to return to the extrapolations from high doses.

The reason for high-LET-radiation–induced LDHRS is complicated. Marples *et al.* [7] reported that LDHRS is reduced and perhaps absent for neutron and pion radiations. However, Bohrnsen *et al.* [22] declared the LDHRS response exists in V79 cells irradiated with 100-MeV/u carbon ions. Xue *et al.* observed LDHRS in GM0639 normal human fibroblasts irradiated with a broad beam of 70-keV/μm carbon ions [23]. In this study, the LDHRS phenomenon was observed at low doses for high-LET carbon ions in terms of not only cell survival but also phospho-histone γH2AX foci and micronuclei formations. To date, LDHRS is more commonly observed in fibroblasts than in epithelial cells or keratinocytes in normal tissue-derived cell lines [10]. Hueskin *et al.* [13] reviewed the data from existing experiments and concluded that the LDHRS mechanisms are identical for sparsely ionizing radiations and densely ionizing charged particles, and LDHRS occurs in a LET-dependent manner. However, based on the microscopic dose and the probability of not being hit calculations, it could be deduced that the probability of the nucleus being hit and of cytotoxic substances spreading from adjacent cells are also factors determining cell fate. Particle type, macroscopic dose, dose uniformity in the radiation field and the activity of GJIC could be influencing factors for high-LET radiation. A series of experiments using X-ray microbeams suggested that the site of exposure in cells is a key variable in low-LET-radiation–induced bystander effects and LDHRS for V79 cells [24, 25]. Briefly, nucleus exposure led to enhanced LDHRS, whereas cytoplasm exposure resulted in enhanced IRR and suppressed LDHRS. The surviving fraction of bystander cells decreased monotonically when whole cells were irradiated. Otherwise, a parabolic enhancement of bystander cell death was observed when only the nuclei were irradiated. These observations imply that the area ratio of nucleus to cell could be another influencing factor for LDHRS in high-LET radiation, because the probability of being hit varies with the area of target. Therefore, the LDHRS phenomenon induced by high-LET radiation is elaborately regulated by these factors.

GJIC is a major mechanism for the propagation of radiation effect factors between cells. Autsavapromporn *et al.* [20], using broad beams of gamma-rays and α particles ( < 100 cGy), reported that α-particle-radiation–induced toxic effects were spread by GJIC, but that this was not so for γ-rays; they also noted that the co-culture time after radiation at a confluent state was also a crucial factor. In another study using X-ray and heavy-ion microbeams, Autsavapromporn *et al.* proved that the bystander effect was mediated by GJIC after exposure to high-LET heavy ions ( < 60 cGy) instead of low-LET X-rays [26]. A GJIC-mediated bystander effect for high-LET radiation is also supported by our results using high-LET carbon ions. Prise *et al.* [27] suggested that GJIC might be governed by cell type and context at the time of irradiation. Due to insufficient data up till now as to whether GJIC works in high-LET radiation, still needs further study.

The present study emphasizes the impact of NTEs on LDHRS for high-LET particle radiation. However, it does not argue against the possible mechanism of the LDHRS phenomenon being contributed by cells at a radiosensitive cycle phase. Xue *et al.* [23] proposed that LDHRS demonstrates ataxia-telangiectasia mutated (ATM)-dependent behaviour in mammalian cells that have been irradiated with heavy ions, and that ATM-dependent ‘early’ G_2_ checkpoint arrest is involved. In addition, ATM was found to act downstream of ataxia-telangiectasia and Rad3-related (ATR) in the DNA damage response signaling of bystander cells [28], implying that both NTEs and cell cycle checkpoints play important roles in the high-LET-radiation–induced LDHRS phenomenon.

## CONCLUSION

In this study, we observed LDHRS phenomena in terms of different bio-endpoints in human normal lung fibroblast MRC-5 cells exposed to high-LET carbon ions at low doses, at which the direct hit effects by carbon ions did not yet dominate. GJIC-mediated NTE was found to be a decisive factor in the LDHRS phenomena, i.e. the NTE could causally contribute to the final outcomes in the low-dose region.

## FUNDING

This work was jointly supported by the Key Project of the National Natural Science Foundation of China (Grant No. U1232207) and the National Natural Science Foundation of China (Grant No. 10905080, Grant No. 11075191 and Grant No. 11205217). Funding to pay the Open Access publication charges for this article was provided by the Key Project of the National Natural Science Foundation of China (Grant No. U1232207).
